# Using virtual worlds as a platform for collaborative meetings in healthcare: a feasibility study

**DOI:** 10.1186/s12913-020-05290-7

**Published:** 2020-05-19

**Authors:** Michael J. Taylor, Chiya Shikaislami, Chris McNicholas, David Taylor, Julie Reed, Ivo Vlaev

**Affiliations:** 1 Department of Surgery and Cancer, Imperial College London, London, SW7 2AZ UK; 2 CLAHRC for North West London, CLAHRC offices, Chelsea and Westminster Hospital, 369 Fulham Rd, London, SW10 9NH UK; 3 Warwick Business School, University of Warwick, Scarman Road, Coventry, CV4 7AL UK

**Keywords:** Virtual worlds, Quality improvement, Multidisciplinary team meeting, MDT

## Abstract

**Background:**

Healthcare teams often consist of geographically dispersed members. Virtual worlds can support immersive, high-quality, multimedia interaction between remote individuals; this study investigated use of virtual worlds to support remote healthcare quality improvement team meetings.

**Methods:**

Twenty individuals (12 female, aged 25–67 [*M* = 42.3, *SD* = 11.8]) from 6 healthcare quality improvement teams conducted collaborative tasks in virtual world or face-to-face settings. Quality of collaborative task performances were measured and questionnaires and interviews were used to record participants’ experiences of conducting the tasks and using the virtual world software.

**Results:**

Quality of collaborative task outcomes was high in both face-to-face and virtual world settings. Participant interviews elicited advantages for using virtual worlds in healthcare settings, including the ability of the virtual environment to support tools that cannot be represented in equivalent face-to-face meetings, and the potential for virtual world settings to cause improvements in group-dynamics. Reported disadvantages for future virtual world use in healthcare included the difficulty that people with weaker computer skills may experience with using the software. Participants tended to feel absorbed in the collaborative task they conducted within the virtual world, but did not experience the virtual environment as being ‘real’.

**Conclusions:**

Virtual worlds can provide an effective platform for collaborative meetings in healthcare quality improvement, but provision of support to those with weaker computer skills should be ensured, as should the technical reliability of the virtual world being used. Future research could investigate use of virtual worlds in other healthcare settings.

## Background

Computer-generated, online virtual environments known as ‘Virtual Worlds’ have been utilized in many different organizational contexts [1–3]. Virtual worlds have been used for healthcare-related teaching [4], dissemination [5] and simulation [1, 6] but their use as a platform for healthcare staff team meetings has not previously been studied. Teams working in healthcare, such as multidisciplinary teams and quality improvement teams often include members based across different hospital sites, making face-to-face meetings potentially difficult for members to attend [7, 8]. Videoconference has been used to enable meetings between staff across hospital sites, saving travelling time and costs [7, 9]. Despite the demonstrated effectiveness of this method [9], and with ongoing improvements in available audio and video quality and greater bandwidths becoming more widely available [10], disadvantages have been cited including loss of richness of information resulting from nonverbal cues, constraining effective communication [9, 11]. It has been suggested that this can result in participants unconsciously using a more assertive communication style, with negative effects on collaborative discussion [11, 12].

A virtual world platform offers an alternative communication method between remote team members. The present study investigated use of virtual worlds as a platform for collaborative communication between members of healthcare quality improvement project teams. Participants’ perceptions of the feasibility and desirability of using virtual worlds in health care communication were measured, as were the quality of team output in virtual world and equivalent face-to-face settings.

Users of virtual worlds interact with the virtual environment, and with one another through digital self-representations known as ‘avatars’ [13]. Users can talk to each other using computer-connected microphones as well as written messages [13]. Virtual worlds could be useful in healthcare contexts when staff or patients meet remotely [14], and when the virtual environment can be used to facilitate discussions or collaborative activities [15, 16]. Documents can be worked on collaboratively within the virtual environment [15], the environment can be modified to represent any form of physical setting [17] and tools for collaboration can be built that would be difficult or costly to represent in equivalent face-to-face meetings [6, 15]. Virtual worlds can give users the impression that they are all sharing the same physical space, resulting in a sense of ‘togetherness’ that cannot be created through video link or any other remote communication method [18, 19].

There may be unanticipated barriers to use of virtual worlds for meetings between healthcare team members. The present pilot study investigated acceptability of use of virtual worlds for health quality improvement project team meetings. Perceptions of the extent of its efficacy, and potential advantages and barriers to its adoption in healthcare by members of the teams who used it were also studied.

### Aims

- Compare quality of collaboration between face-to-face and virtual world settings by comparing how successfully participants completed a task in each setting
- Investigate participants’ perceptions of using a virtual environment and the collaborative task they completed within it
- Investigate attitudes of participants towards use of virtual worlds for group collaboration in healthcare

## Method

### Study design

The experiments described here involved established teams who worked on healthcare quality improvement projects undertaking collaborative problem-solving and reporting tasks. Teams of participants either conducted the tasks in a virtual world, or in a face-to-face setting. The goal of the task was to undertake a problem-solving activity and to record this collaboratively using an Quality Improvement method known as ‘Plan-Do-Study-Act’ (PDSA) [20]. The PDSA method was originally developed in the manufacturing industry, and has been utilized in healthcare settings with the aim of facilitating improvements in service delivery [21]. It is a 4-stage learning cycle, which aims to structure tests of change to complex systems. The changes are adapted iteratively as users learn what works and what does not. The test start at a rapid, small-scale; this can increase if confidence of success grows [20, 22]. The method promotes a systematic approach and allows users to examine and react to the effects of changes [20], so it can be a useful tool in healthcare improvement and research [23, 24]. There is evidence, however, that the method is not always optimally used and it has been suggested that the method is more complex than many of its users realize [25, 26]. Using PDSA cycles in a team is a task that requires collaborative negotiation and decision-making at each of the four stages.

All participants had received equivalent training in using the PDSA method through their shared affiliated research network and all had experience of reporting PDSA cycles as part of their healthcare quality improvement work. Members of the teams included clinicians, academics and technicians and the teams worked on projects that aimed to improve quality of healthcare delivery across multiple health sectors. There were several dimensions of quality that can be assessed in the conduct of PDSA cycles [23–25], and reporting these cycles collaboratively in relation to the problem-solving task requires a high standard of team communication. The problem solving task used in this test was the ‘Towers of Hanoi’ (ToH) task, which has been demonstrated to be useful for studying collaboration [27]. The ToH task involves moving discs between 3 poles in a certain order. A non-healthcare related task was used so that the variety of healthcare skills and experience of participants would not affect results. The goal and rules for ToH are simple, but completion is highly challenging [28]: distinct steps of moves must be made, so effectively reviewing progress whilst in the process of completing the task is a useful tactic [29]. A time constraint was used in this study to prompt participants to consider their strategy.

Interpersonal collaboration is influenced by perceived power and group hierarchy [30, 31]. Academic and clinical organizational environments tend to have clearly defined hierarchical structures that are indicative of an individuals’ level of power, according to their professional role [32, 33]. Findings from negotiation research suggest an advantage in negotiation outcomes for traditionally low-power roles in computer-mediated communication, compared to face-to-face communication [34]. An effect of leveling established hierarchies and empowering people of traditionally lower-power roles could aid collaborative working in multidisciplinary teams in traditionally hierarchical organizational settings such as healthcare [35]. Participants were asked the extent to which they felt they had power in discussions with their team to investigate whether these perceptions were difference between real and virtual world conditions.

The primary outcome of the present study was the extent to which the virtual world successfully provided a platform for the collaborative task. The reported PDSA cycles were assessed to see if there were differences in quality of conduct between those constructed in virtual world and face-to-face settings. The face-to-face condition was primarily used to determine baseline levels for PDSA task outcomes and perceptions of power; this is why four teams were recruited to take part in the virtual world condition whilst only two were recruited to take part in the face-to-face condition. Participants of the virtual world condition reported the extent to which they felt immersed in the collaborative task and in the virtual environment. A subset of participants of this condition also took part in semi-structured interviews to gain in-depth qualitative data on their perceptions of using the virtual worlds.

### Participants

This study was granted ethical approval by the relevant institutional committee. Informed written consent was obtained from all participants. Twenty individuals (12 female) aged 25 to 67 (*M* = 42.3, *SD* = 11.8) took part. All were professionals affiliated to one of six multidisciplinary teams who worked on healthcare quality improvement projects (see Table 1) based in London. Every participant had experience of using PDSA methodology as part of their team project work. All participants gave informed consent to take part in the study, which was approved by the relevant institutional ethics committee.

**Table 1:** Table depicting roles in teams who took part

Team	Condition	Number of members with job role		
		Doctor	Nurse	Non-clinical Academic / technician
1	Virtual world	1		2
2	Virtual world	1	3	
3	Virtual world	1	1	
4	Virtual world	1	1	1
5	Face-to-face	2		3
6	Face-to-face	1	1	1

## Materials

### The virtual world

The virtual world used was Second Life (secondlife.com). Second Life was selected because it is publically accessible, free to use, and has been used for healthcare training [6, 36] and education.

### Towers of Hanoi task

**Fig. 1:**
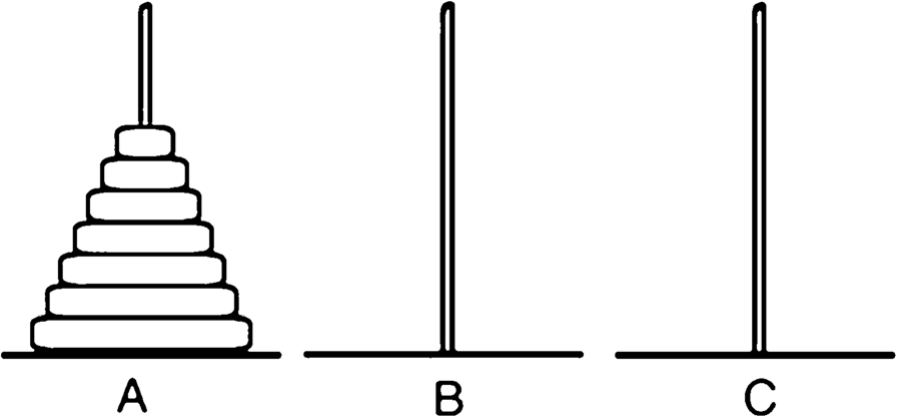
Diagram showing the starting positions of the Towers of Hanoi task

The ToH task consists of 3 poles on which 7 hollow disks are placed. At the start, all 7 disks are stacked on 1 pole (pole A), in order of size with the largest disk on the bottom of the pile and the smallest disk on the top (See Fig. 1**)**. The aim of the task is to stack as many of the disks on a different pole (B or C) as possible. Only 1 disk can be moved at a time and that larger disks cannot be placed on top of smaller disks. The task can be completed in a minimum of 127 moves.

### PDSA reporting

In the face-to-face condition, A2 sheets of paper and an assortment of pens were presented to participants, who were told that they could use these materials to document the PDSA cycles in any way that they liked. Participants in the virtual world condition used a purpose-build virtual reporting tool. This consisted of a board displaying a document, upon which users could type simultaneously (see Fig. 3). Text was automatically highlighted in different colors, depending upon the identity of the writer (see Fig. 3). This tool was supported by an external website (titanpad.com).

### Presence questionnaires

Presence is a measure of the extent to which an individual experiences a virtual environment as being real [37]. It is an important factor that can influence collaboration between virtual world users [38] ‘Collaborative Virtual Presence’ (CVP) measures the degree to which individuals in a virtual world feel as though they are collaborating in a real environment [39]. Four CVP subscales were measured here: Absorption (the extent to which individuals were absorbed in the task), Immersion control (the extent to which individuals felt in control of their virtual experience), Immersion sensory (The extent to which participants were able to visually sense the virtual environment) and ‘Awareness’ (extent of use of nonverbal cues).

Participants in the virtual world condition completed Presence and Collaborative Virtual Presence (CVP) questionnaires, developed by Fox and colleagues [37] and Massey et al. [39] respectively. Answers were on a 7-point Likert scale from 1 = Strongly Disagree-7 = Strongly Agree.

### Perceived power

Participants of the collaborative tasks were asked ‘How much power did YOU feel that you had in the discussions?’ and responded using a 7-point Likert scale, ranging from 1 = Not much to 7 = A lot. The purpose of this was to examine whether job role affected the extent that participants’ perceptions of their own power when communicating with others during the collaborative task.

### PDSA guidelines

Each participant was provided with a document explaining recommended PDSA cycle reporting and conduct according to healthcare research literature [25]. This was to ensure all participants had a recent reminder of best practice recommendations before they commenced the task, as there is evidence that the PDSA technique is frequently not fully adhered to [25]. They were advised to follow the advice in the document when they reported their PDSA cycles.

### Procedure

#### Face-to-face setting

Participants sat at a table together, on which there was a small (18 × 8 × 6.5 cm) wooden ToH task.

#### Virtual world setting

**Fig. 2:**
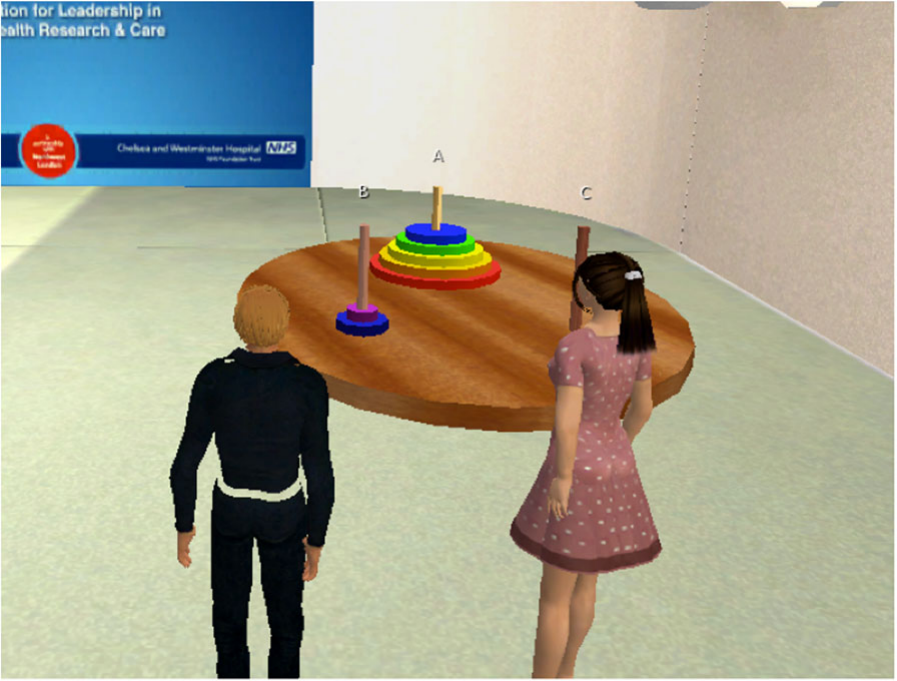
Avatars, representing two participants, in front of the virtual Towers of Hanoi game

**Fig. 3:**
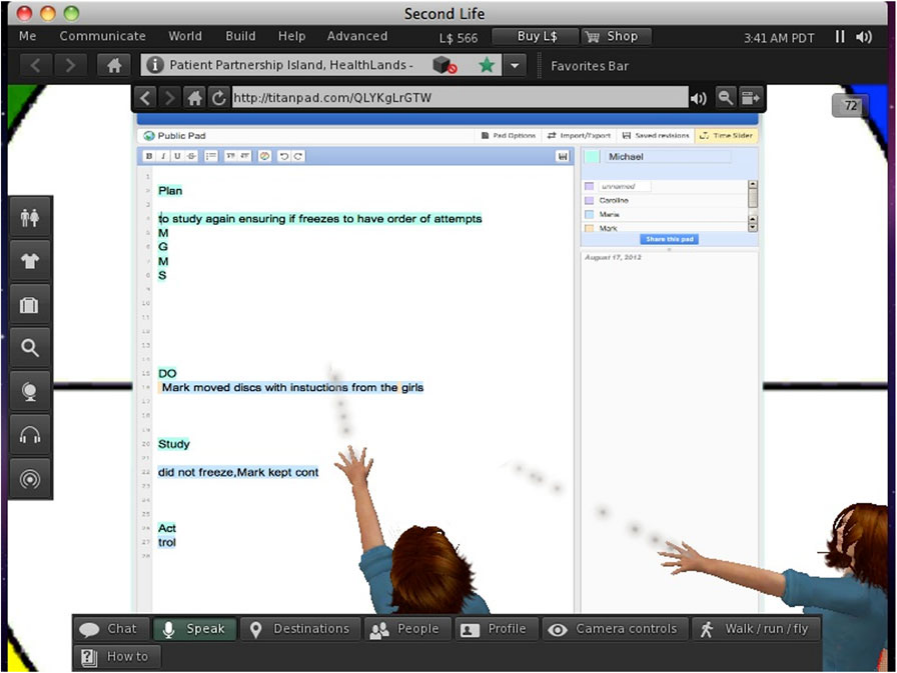
Two participants’ avatars using the reporting tool to document a PDSA cycle together

Prior to commencing the task, participants attended a session where a presentation was given about Second Life and how to use it. They then, before the task, were each given instructions on how to use Second Life, and a member of the research team was available to explain to each participant individually how to use the software to move their avatar, change their view, and interact with the other avatars through text, voice, or both, and how to interact with the towers of Hanoi task objects. Participants were then positioned in separate rooms and each accessed Second Life using a laptop computer. Participants had generic, gender appropriate avatars randomly selected for them. Within Second Life, participants’ avatars were positioned in the same virtual meeting room. A virtual depiction of the ToH (see Fig. 2) was represented in this virtual world room along with a tool to be used for documenting the PDSA cycles (Fig. 3).

#### Procedure for both conditions

Participants were given a total of 30 min to attempt to progress as much as possible on the ToH, whilst completing high-quality PDSA reports. At the start of the session, participants spent 2 min examining the (physical or virtual) ToH representation, then were asked to complete the ‘Plan’ stage of the cycle. For the next 2 min they were allowed to move the ToH discs. They then completed writing the report for the remainder of the PDSA cycle and produced a Plan for the next cycle. They then spent a further 2 min moving the ToH discs before reporting the remaining 3 stages of the 2nd PDSA, then next Plan for the 3rd stage. They continued in this manner for the duration of the task.

#### Analytic strategy

The quality of reported PDSAs, interview data, subjective immersion in the virtual world and perceived power were the study outcome variables.

Semi-structured interviews were conducted with 5 participants from the virtual world conditions (one doctor, two nurses and two non-clinical health researchers) with every team from this condition being represented by at least one person. An interview guide was created for this study (please see Supplementary file 1). Participants were asked about their experience of using the virtual world. Questions related to participants’ perceptions of the communication between members of their team, what it was like using the virtual world and of potential for virtual worlds to be used as a platform for collaborative healthcare quality improvement project meetings. Interviews were audiotaped and transcribed. Thematic analysis [40] was conducted by 2 researchers who independently identified themes, which were developed into a framework for coding the body of data. Multiple coding tested the acceptability and reliability of designated categories.

Quality of reported PDSA were assessed using a theoretical framework, which was recently developed to identify heterogeneity between PDSA cycles reported in the research literature [25].

## Results

### Using virtual worlds for PDSA reporting

Four project teams attempted to report PDSA cycles in virtual worlds. Three did so with little or no difficultly. One team (Team 4) was unable to report their PDSA cycles due to a fault that resulted in the external website supporting the virtual reporting tool being inaccessible.

Temporary technical problems, due to the voice server, were experienced by 2 teams (Teams 1 and 2), but these were resolved within 5 min in each occasion.

### PDSA reporting

Table 2 displays the analysis of the team’s written PDSA reports (Team 4 were unable to use the virtual PDSA reporting tool). Most of the key components of the PDSA cycles were successfully reported by the teams; one component that the teams consistently failed to report was the making of a prediction in the ‘Plan’ stage of the PDSA cycle.

**Table 2:** Analysis of PDSAs reported during the task (virtual world = Virtual world condition, face-to-face = face-to-face condition. Team 4 were not able to report PDSA cycles due to a technical fault)

PDSA Feature under examination	Team / Condition				
	1 virtual world	2 virtual world	3 virtual world	5 face-to-face	6 face-to-face
1. Were multiple cycles used?	Y	Y	Y	Y	Y
2. Were multiple cycles linked to one another (i.e. does the Act stage of one cycle inform the Plan stage of the cycle that follows)?	Y	Y	Y	Y	Y
3. Was a change tested?	Y	Y	N	Y	Y
4. Was an explicit prediction articulated?	N	N	N	N	N
5. Has the application of PDSA method been detailed in the reports?	Y	Y	Y	Y	N

### Presence results

Participants tended to experience low levels of Presence (M = 3.3, *SD* = 1.7). The mean global CVP score was 3.7 (*SD* = 1.3). The subscale scores were highest for ‘absorption’ (*M* = 4.9, *SD* = 1.7), followed by ‘Immersion Control’ (*M* = 3.7, *SD* = 1.6), then ‘Immersion Sensory’ (*M* = 3.6, *SD* = 2.0) being similar to one another and slightly lower than absorption and ‘Awareness scales’ receiving the lowest scores (*M* = 1.8, *SD* = 1.4).

### Perceived power and job role

Participants’ jobs were coded as belonging to 1 of 3 categories: Doctors (*N* = 7), Academics and Technicians (*N* = 7), or Nurses (*N* = 6) (See Table 1). A Kruskal-Wallis test revealed job category to have a significant impact upon the extent of power that participants reported feeling (*X*^*2*^ [2]= 6.34, *p* = .041), with Doctors tending to feel that they had the most power (*M* = 5.7, *SD* = 1.0), followed by Academics and Technicians (*M* = 5.0, *SD* = .9), with Nurses feeling that they had the least power (*M* = 3.8, *SD* = 1.2). Perceived power was not dependent on whether participants were in the virtual world of face-to-face condition (*F* [2, 18] = .551, *p* = .59).

### Interview results

Interviews lasted from 15:18 to 32:56 min (M = 25:56, SD = 7:22). Three major themes: ‘collaborative working in a virtual environment’, ‘Remote electronic organizational interaction’ and ‘Suggestions for future research’ were identified. All comments relating to advantage or barriers of virtual worlds were collated and are summarized in Table 3.

**Table 3:** Summary of drivers, barriers and possible actions regarding use of virtual worlds (virtual worlds) for healthcare meetings identified at interview

	Results from semi-structured interviews	Possible actions/solution regarding results
Advantages of using virtual worlds for healthcare meetings	• Use of virtual world for remote meetings would save travelling time • Some people find it easy to use (possibly people who are used to playing computer games and who have good computer skills) • Using virtual worlds can be enjoyable • There have been many technological advances in communication so people may become used to using virtual worlds one day if their use becomes more widespread • The ability to use the virtual environment to support collaborative activities means there is great potential for developing new ways for these meetings to be facilitated • A virtual world could be used to manage group dynamics and interaction in virtual setting • A virtual world can be used to provide clarity and structure to tasks that are traditionally seen as complex when used in the ‘real world’ • virtual worlds can be used to explore ‘risky’ behavior in safe environment • It is possible to embed tools within environment that can facilitate virtual world meetings • Use of virtual worlds for meetings could result in people who are nervous when meeting face-to-face gaining confidence and being more vocal • virtual worlds could be used to host focus groups where attendees remain anonymous and therefore are able to express their opinions more freely	• virtual worlds facilities could be provided as an alternative to remote meetings • Better usability of virtual worlds will mean higher engagement. It may be advantageous to initially test usability of those who already have high computer skills when studying behavior in virtual world settings. • virtual world facilities could be designed to be fun to use in an effort to engage users. Widespread use of virtual world would be helpful in engaging teams to use virtual world facilities as the concept would be more familiar to more people • There is huge potential for using the virtual environment to enhance group communication, which can be tested through future research • This could be explored in future research. It may be useful to investigate group dynamics in virtual world settings in greater depth before investigating ways in which group dynamics can be altered and improved. • virtual worlds could be used in education through simulation of tasks. This may be particularly useful for teaching group exercises. • This is a further reason why virtual world may be useful for providing educational simulations • This is a potentially important advantage of virtual worlds that can be explored through future research • virtual world meetings may be useful in empowering representatives of traditionally low-power groups, which may be advantageous in some contexts. • virtual world may be useful for collecting data on sensitive subjects where participants may be otherwise deterred from contributing.
Barriers to using virtual worlds for healthcare meetings	• Many are unfamiliar with using virtual world software, making it potentially difficult for this sample to use it • Using virtual worlds is potentially difficult for people with low computer skills who work in healthcare. • Lack of non-verbal communication makes people more difficult to read because you can’t see their facial expressions and other non-verbal nuances are lost. Furthermore it is not possible to engage in tactile communication when using virtual world, which can result in more emotionally distant communication • Attendees of virtual world meetings may be distracted by the content of the meeting by the technology • Technical difficulties can be distracting. There is a danger of attendees missing information due to technical faults. • It can take time and resources to train people to use virtual world software	• Technical support would help users to know how to address technical problems with software. Also, a more user-friendly virtual world than Second Life might get developed in future. • Technical support may alleviate this affect somewhat. Also, it may be best to initially recruit users who are already comfortable and confident with using computers. • This is an important potential disadvantage of using virtual worlds. For certain purposes, they may never be as useful as face-to-face communication, but they might provide a highly useful remote-communication option. • The virtual environment in virtual meeting rooms could be designed with the intent of directing user’s attention towards the speaker or the content of the meeting. Technical difficulties can certainly be problematic with this software. It might be that virtual world software becomes more reliable over time. Procedures could be put in place so that attendees of meetings are able to alert someone immediately if they feel they might have missed information in the meeting. This is an important consideration if virtual worlds might be used for important healthcare meetings. • Comprehensive instructions and checklists could be provided online by a central body. Additionally initiatives could be provided for members of teams with good computers skills to teach others in their team how to use virtual world software

#### Collaborative working in a virtual environment

Amongst the 5 interviewees who were in the virtual worlds condition, there were a variety of opinions on how difficult Second Life was to use. One participant reported Second Life as being easy to use, 2 reported it as being mostly easy to use, but highlighted challenges relating to directing other users’ attention to objects in the virtual room, and 2 reported it as being challenging and that they would like training if they were to use it again. It was suggested that the varying levels of potential users’ confidence with the technology could affect team interaction in virtual worlds. Two participants stated that they enjoyed using the virtual world. One suggested that virtual worlds might have more appeal to younger adults, as they tend to be more knowledgeable about using computer technologies.

Most participants stated that the main difference between communicating face-to-face, and using a virtual world is the lack of non-verbal communication and eye contact in the latter context. Some cited this as a disadvantage, because it restricts nonverbal communication, but it was also suggested that limited non-verbal communication – and therefore fewer social distractions - could be beneficial for teamwork, and could result in virtual world users being more task-focused.

#### Remote electronic organizational interaction

Participants were asked if they thought that interactions between members of the teams in a virtual world context would be different to equivalent face-to-face interactions. Diverse opinions were elicited. Comments were made suggesting that interaction would be similar across contexts:

***Participant 1:****“So we’re having a meeting in Second Life and there’s four of us all in different places, okay, and I would imagine that, because there’s a blank sheet to start with, we might feel okay about just all chipping in. But then, if we’re kind of getting towards the final outcome, that what is our blank piece of paper finally going to look like at the end, I would suspect that we would always end up bowing to the, bowing to the consultants”.*

But there some thought that a virtual world context would result in a change in interactions, especially those affected by traditional hierarchical structures:

***Participant 5: “****Well, it should break down hierarchy because people that are nervous might have more willingness to participate. … the quieter ones might feel less nervous to talk, and I think more to the point, if you use it within teams that haven’t met before, you might get a different personality emerging from that person.”*

Some participants suggested that, as the team members already knew each other and were already immersed in the organizational culture, those with traditionally low-power roles might feel more confident in voicing their opinion, but there may not be much change in interaction overall.

One participant described a prior experience where they had negotiated with two other people using a voice-conferencing tool. They felt that restrictions on non-verbal communication of this mode resulted in a less-favorable outcome than an equivalent face-to-face negotiation would have produced.

***Participant 1:****“I think I had no sense of where the next question was going to come from perhaps. So you’ve got two people … … it came as being both of them at once, whereas if they’d been separate, if I could see them separately, I might have felt able to respond to that one and then get my thoughts together and then respond to that one. But they felt merged”.*

#### Ideas for further virtual worlds research

Some participants put forward ideas for how virtual worlds could be used in healthcare in the future. Suggestions were made that virtual worlds could be used to provide a platform for delivery of clinical training and Multidisciplinary Team Meetings [this type of meeting involves a group of doctors and affiliated health professionals, who discuss and manage the care of patients with complex medical conditions]). One participant suggested that virtual worlds could host virtual meeting environments with features than discourage group decision making biases that can hamper face-to-face meeting, such as ‘group think’ [41].

***Participant 3*:** “*In the real world certain teams, I think they’d get bogged down in various, well, various social psychological phenomena which I’m sure you’re familiar with; group think and other forms of social biases may operate. And I can see that it would be possible to design access rights to interaction and to airtime within the virtual environment in such a way that would mitigate negative effects of those things, or whatever, from occurring*”.

One of the interviewees commented that they had found the PDSA task useful because it helped them to better understand PDSA method. They suggested that virtual worlds could be used as a platform for teaching PDSA method.

***Participant 4:****“If you’d been given a problem and said, use the PDSA cycle to solve the problem, that would have been much more straightforward than all the complex description that went on with it and the confusion. And actually, at the time, I thought they should have just said, you know, as a group, here’s a PDSA, here’s a problem, use the PDSA cycle as a way of teaching how to do it, not gone into all that descriptive language.”*

## Discussion

Quality of collaborative task outcomes was high in both virtual world and face-to-face conditions. Interview data indicated multiple perceived advantages of using virtual worlds in healthcare and suggestions for other healthcare contexts where their use could be implemented, as well as possible barriers to successful adoption that would need to be considered. However, some technical problems were experienced; if virtual world were to be used for important healthcare meetings, it is crucial that participants are able to communicate effectively at all times, as missed or misunderstood communications could have catastrophic consequences, especially in meetings about patient management such as Multidisciplinary Team Meetings. The majority of participants in virtual world conditions found the virtual world of Second Life easy to use, though some found it difficult. It seems, therefore that implementation of a virtual world platform to support meetings in healthcare would require significant technical support for individuals using the system. There is evidence that in some cases failing to persuade all members of a quality improvement project to attend meetings can be a significant barrier to success of the project [42], and if the virtual world platform was seen as a factor that made meetings more difficult, this could worsen attendance further. It should be noted that an in-depth exploration of usability issues was not carried out in this study, which is a limitation, and future work could address this.

Job roles predicted individuals’ perceptions of their own power across conditions. There were no differences in perceived power between conditions, though interview data revealed a variety of opinions on the extent to which a collaborative virtual world setting might affect traditional power structures. Research has indicated that conducting negotiations remotely, using electronic communication, can alter the way that power structures influence negotiation outcomes [34], and research has also shown other factors specific to virtual worlds such as avatar appearance to effect negotiation behaviors [43], and such potential effects should be considered when using virtual worlds in healthcare related communication.

Presence scores and CVP revealed a tendency for participants to not experience the virtual environment as being ‘real’, but suggest they felt absorbed in the tasks they performed in the virtual environment. This is consistent with other research suggesting Second Life to induce relatively low levels of presence [44]. Alternative virtual worlds that enable a greater degree of non-verbal communication are likely to be more effective inducing greater levels of presence, which in turn may increase quality of collaboration [1, 38].

A participant suggested possible use of virtual worlds to host Multidisciplinary Team Meetings provides an example of an alternative multidisciplinary collaborative health setting that could benefit from provision of a platform for high-quality remote interaction [45, 46]. This is a context where the quality of shared decision making is crucial and can be affected by inter-personal team dynamics [35]; future work could investigate the extent to which a virtual world platform affects these dynamics. The virtual world could also be designed in such a way as to intentionally influence group dynamics in a positive way that could aim to, as suggested by a participant, reduce group decision-making biases such as ‘group think’ (a collective desire for cohesion and unanimity resulting in groups making exceptionally poor decisions [41, 47]).

Interview results suggest conducting and reporting PDSA cycles in a virtual world can potentially help to make the PDSA method easier to understand. Doing a simulated task in a virtual world might allow people to comprehend features of the task that they previously not noticed or had become habituated to, because they are experiencing it in a new context; the virtual world PDSA representation might have helped to simplify and compartmentalize the PDSA procedure, making it easier to understand. An important way that virtual worlds have been used in education, apart from as communication spaces [48, 49] and simulation of real-world spaces such as university campuses [50], is as an ‘experiential spaces’ [51], where users can manipulate and interact with objects to help them to learn. The interactive PDSA tool appears to have been used as an experiential learning space in the present study.

Results of the interviews also indicate that the virtual world context might have encouraged participants to take greater care in ensuring clarity in their verbal and written communication, so that roles were allocated appropriately and attention was focused on relevant stimuli.

A limitation of this study was the lack of a videoconferencing arm of the experiment. It would be of benefit to compare collaborative teamwork in virtual worlds to teamwork conducted across videoconferencing to compare relative perceptions of quality of interaction and collaboration, as well as presence. Future work could investigate such a comparison. Advantages of using videoconferencing over virtual worlds include that training may be less likely to be necessary.

Studies investigating collaboration in 3-dimensional virtual environments have investigated nonverbal communications [1, 38] and have revealed evidence for collaborative presence to be influenced by factors such as avatar gestures [1]. This was not investigated in the present study, and it may be beneficial for future studies to investigate the influence of nonverbal communication on team meetings in healthcare and the impact this may have on presence and collaborative work.

There was variability in the number of participants in the different teams, and this is likely to have affected the groups’ communication and collaborative working. Whilst in this feasibility study, this enabled a greater range of group sizes to be observed, potential future work that aims to gain generalizable data on collaborative work should perhaps focus on groups of a certain size.

The general high standard of PDSA reporting across conditions demonstrates the potential for complex collaborative tasks to be carried out in virtual worlds. Because of this ‘ceiling effect’, as well as the small sample size, generalizable differences in PDSA reporting between face-to-face and virtual worlds conditions could not be inferred. However, across both conditions, teams failed to elicit a prediction in the Plan stage. Use of predictions is an important feature of the method [25], and many PDSA cycles described in health services literature fail to elicit predictions as part of the cycles they describe [25]. A virtual world PDSA reporting tool could be adapted to encourage users to enter a hypothesis in the Plan stage, which would improve quality of reported cycles.

## Conclusions

Virtual worlds can be used to support remote collaborative meetings in healthcare, with potential to be used in the place of face-to-face meetings, or meetings that take place by videoconference. The interviews identified a number of important advantages and barriers (See Table 3) and possibilities for future research. Second Life might be suitable for providing a platform for healthcare quality improvement team meetings. Investigation of use of virtual worlds for an applied healthcare team decision-making task such as an Multidisciplinary Team Meeting may also be a useful avenue of investigation, though it would be necessary to use a different virtual world for this purpose, due, at least in part, to the need for a platform that is highly reliable in supporting verbal communication. Investigations into how Multidisciplinary Team Meeting decision making can be quantitatively analyzed [52] could provide outcome variables for such work.

## Supplementary information

**Additional file 1.** Interview guide.

## Data Availability

Data and materials are available upon request from Michael J Taylor at michaeljtaylorresearch@gmail.com
