# Supplementary material for: Using virtual worlds as a platform for collaborative meetings in healthcare: a feasibility study
Source: BMC Health Serv Res. 2020 May 19;20:442. doi: 10.1186/s12913-020-05290-7 (PMC7236942; doi:10.1186/s12913-020-05290-7)
Supplement: Supplementary file 1 — Additional file 1. Interview guide. [file 12913_2020_5290_MOESM1_ESM.docx]

**Interview guide**

1. Could you introduce yourself and give us a brief overview of your job role and your team’s project
   - What is your role in the team?
2. How many of you are there in the project team?
3. The members of the team present in the virtual worlds experiment were ________ what are their roles in the team?
4. How frequently do the ____ of you meet?
   - How long is this for?
5. What communication modes do you use to interact with your team (other than face-to-face)?
   - How often do you use these modes compared to face-to-face
6. Would you say there is a clearly defined hierarchical structure in the team?
7. Have you used PDSA quality improvement methodology before?
   - How useful is it for conducting and reporting health research?
8. What are your views regarding use of the PDSA quality improvement method for your team’s project?
9. Could you describe the experience of using the virtual world of Second Life for conducting a collaborative activity?
10. What do you think of the prospect of collaborative reporting of PDSA cycles in a virtual environment?
11. Do you have any further comments regarding the prospective use of a virtual world for team meetings or for conducting collaborative activities in health research?
12. Any other comments regarding anything to do with the study or anything that we have mentioned?
